# Clinical outcome of standardized ^177^Lu-PSMA-617 therapy in metastatic prostate cancer patients receiving 7400 MBq every 4 weeks

**DOI:** 10.1007/s00259-019-04584-1

**Published:** 2019-11-28

**Authors:** Sazan Rasul, Marcus Hacker, Elisabeth Kretschmer-Chott, Asha Leisser, Bernhard Grubmüller, Gero Kramer, Shahrokh Shariat, Wolfgang Wadsak, Markus Mitterhauser, Markus Hartenbach, Alexander R. Haug

**Affiliations:** 1 Department of Biomedical Image-guided Therapy, Division of Nuclear Medicine, Medical University of Vienna, Vienna, Austria; 2 Department of Urology, Comprehensive Cancer Center, Vienna General Hospital, Medical University of Vienna, Vienna, Austria; 3 Department of Urology, Weill Cornell Medical College, New York, NY USA; 4 Department of Urology, Second Faculty of Medicine, Charles University, Prague, Czech Republic; 5 Institute for Urology and Reproductive Health, I.M. Sechenov First Moscow State Medical University, Moscow, Russia; 6 Department of Urology, University of Texas Southwestern Medical Center, Dallas, TX USA; 7 CBmed GmbH, Center for Biomarker Research in Medicine, Graz, Austria; 8 Ludwig Boltzmann Institute Applied Diagnostics, Vienna, Austria; 9 Christian Doppler Laboratory for Applied Metabolomics (CDL AM), Medical University of Vienna, Vienna, Austria

**Keywords:** PSMA therapy, PSMA PET, Prostate cancer, mCRPC, PSA response

## Abstract

**Purpose:**

[^177^Lu]Lu-PSMA-617 radio-ligand therapy (PSMA-RLT) is emerging in patients with an advanced metastatic castration-resistant prostate cancer (mCRPC). Here, we aimed to estimate the results of PSMA-RLT in terms of response, progression-free survival (PFS), and overall survival (OS) in patients receiving a highly standardized treatment regimen due to mCRPC. The toxicity of PSMA-RLT has also been evaluated.

**Patients and methods:**

Fifty-four patients (mean age 72 ± 7 years, median PSA at time of initial therapy 66 [range 1.0–4890 μg/L]), receiving three PSMA-RLT cycles (mean 7315 ± 573 MBq) at four weekly intervals, were included in this retrospective analysis. Hematological and biochemical parameters were regularly determined in every patient. Kaplan-Meier estimates were used to assess PFS and OS and a Cox proportional hazard model was used to analyze significant associations. Treatment response was based on PSA measurements 4 weeks after the 3rd treatment.

**Results:**

The majority of patients were previously treated with abiraterone/enzalutamide (69%) and docetaxel/cabazitaxel (67%). In total, 79% of the patients showed a decrease in PSA (median PSA decrease from 66 to 19.8, range 0.7–4563 μg/L, *P* < 0.001) 1 month after the 3rd therapy cycle. Among them, 58% and 35% demonstrated a PSA-decline of > 50% and > 80%, respectively. Median OS was 119 weeks; median PFS was 25 weeks. Patients presenting with a PSA decline had significantly longer PFS (27 vs. 15 weeks, *P* < 0.0001) and OS (median survival not reached vs. 52 weeks, *P* < 0.001) than patients with no PSA reduction. Moreover, patients with reduction in PSA levels ≥ 50% (median survival not reached vs. 52 weeks, *P* < 0.0001) and ≥ 80% (median survival not reached vs. 87 weeks, *P* = 0.008) lived significantly longer. While hemoglobin did not change during treatment, levels of platelets (236 ± 71 g/L vs. 193 ± 67 g/L) and leucocytes (6.5, range 2.9–13.7 g/L vs. 4.8, range 1.5–12.3 g/L) decreased significantly, both *P* < 0.001. Two grade 3 leukocytopenia and one grade 3 anemia were observed.

**Conclusion:**

Intense PSMA-RLT regime with four weekly intervals between the cycles is well-tolerated and offers favorable response rates, PFS, and survival rates for patients with mCRPC.

## Introduction

It is estimated that prostatic cancer is the second most common type of cancer and the third leading cause of death in males [1]. The 5-year survival rate among these patients depends on the stage of the tumor and varies from 100% at early stage of the disease to only about 30% in case of presence of advanced metastasis [2]. Over the last few years, the prostate-specific membrane antigens (PSMA), which are enzymes and receptors on the surface of prostatic cells and also known as glutamate carboxypeptidase II, have widely attracted attentions as a target for imaging and treating patients with prostate cancer. Expression of PSMA is strongly increased up to thousand-fold in prostatic cancer and particularly in aggressive as well as metastatic and hormone-resistant prostate cancer. Therefore, [^68^Ga]Ga-PSMA ligand positron emission tomography (PET) is nowadays available and expansively used as a sensitive non-invasive diagnostic method to image prostate tumor and its related metastasis. In addition, [^177^Lu]Lu-PSMA-617 radio-ligand therapy (PSMA-RLT) offers an emerging therapy in patients with an advanced metastatic castration-resistant prostate cancer (mCRPC) [3].

Since the rise of PSMA-RLT, many studies could demonstrate its effectiveness and safeness in mCRPC [4–8]. In a metanalysis of 17 studies with collectively 744 mCRPC patients, PSMA-RLT was commonly well-tolerated by the patients and was associated with only mild degrees of hematological and nephrological toxicities [9]. However, with the exception of the Hofmann et al. study, where all patients received 7500 MBq PSMA-RLT per cycle at 6-week intervals [10], most of the other reported studies in this metanalysis did not have a standardized treatment protocol for PSMA-RLT in mCRPC and the treated patients have received the therapy either as a single dose or in multiple cycles at 8–12-week intervals. Moreover, the treatment plans in these studies were heterogeneous even in terms of therapy activity, as the activity of [^177^Lu]Lu-PSMA ranged from about 1.1 to 9.3 GBq per cycle [9]. So far, it seems there is no established therapeutic protocol concerning PSMA-RLT in mCRPC patients and this might complicate the assessment of the potential of this therapy. Hence, the objectives of this retrospective study were to evaluate rate of response and therapeutic impact of the PSMA-RLT on the progression-free survival (PFS) and overall survival (OS) in mCRPC patients that were all treated with a highly standardized regime and in regular short cycle intervals. Furthermore, we aimed to determine degrees of hematological toxicities associated with the therapy in these patients.

## Patients and methods

### Patients

This retrospective study included all patients, who have been referred to the Department of Nuclear Medicine, Medical University of Vienna, Vienna General Hospital, between September 2015 and September 2018 for receiving PSMA-RLT due to mCRPC. The median duration of follow-up for patients included in this study was 24 months and ranged between 6 and 40 months. All patients were discussed in an interdisciplinary tumor board with the recommendation of PSMA-RLT. The treatments were conducted according to §8 of the Austrian pharmaceutical law (AMG). We have included all patients who were intended to receive three treatments with about 7400 MBq [^177^Lu]Lu-PSMA-617 every 4 weeks. Prior to therapy, a [^68^Ga]Ga-PSMA whole-body PET scan was performed for all patient to illustrate presence of PSMA-overexpression in all lesions [11]. Patients with PSMA-negative metastases have been excluded from this study (*n* = 2). During each hospital admission, patients subjected to a proper physical examination by an experienced medical doctor and, accordingly, Karnofsky-Index und Eastern Cooperative Oncology Group (ECOG) Status, were determined for every patient. Routinely measured laboratory parameters including complete blood counts, biochemistry, and PSA levels were regularly measured for every patient in each hospital visit and 1 month after the last 3rd therapy. Toxicities were assessed following the Common Terminology Criteria for Adverse Events (CTCAE), version 4.0. Furthermore, another [^68^Ga]Ga-PSMA whole-body PET scan was done for all patients 4–6 weeks after the last PSMA-RLT to evaluate and imaging therapy efficacy [11]. The Ethics Committee of Medical University of Vienna has approved the study (EK: 1143/2019) and a written informed consent was provided from patients prior to each therapy.

### Lu-PSMA RLT regime

The therapy was composed of 3 cycles of an intravenous administration of [^177^Lu]Lu-PSMA-617 that has been acquired from ABX GmbH (Radeberg, Germany) and prepared according to methods that have previously been described [4]. In all patients, the interval between each cycle was 4 weeks. During each therapy, patients obtained 1-l normal saline infusion at 300 ml/h, 30 min before administration of an average of 7315 ± 573 MBq PSMA-RLT. Moreover, to protect salivary glands from the effect of the therapy, every patient received prophylactically cool-packs, which have regularly been changed, on their salivary glands 30 min before and up to 6 h after injection of the radiopharmaceutical.

### Statistical analysis

The software IBM SPSS Statistics version 24.0 was used for all data entry and analysis. Prior to any analysis, all data were subjected to Kolmogorov-Smirnov test to determine their distribution. Normally distributed data were expressed as mean ± standard deviation, whereas not normally distributed data were presented as median and range and were log-_10_ transformed for analysis. Categorical variables were shown in percentages and number of reported cases. For comparisons of data between patients responded to those not responded to the therapy, an independent-samples *t* test was used. Kaplan-Meier estimates and a Cox proportional hazard model were used to assess PFS and OS in all patients. PFS was indicated as the time from the first therapy to the detection of the PSA progression and OS was defined from the date of first cycle to the date of the death or to the date of the last hospital visit. In all analysis, a value of *P* < 0.05 was considered statistically significant.

## Results

Collectively, 54 patients were included in this study. Clinical characteristics of these 54 patients (aged 72 ± 7 years, weight 82 ± 13 kg) are shown in Table 1. Thirty-three percent and 67% of the patients presented with a Karnofsky score lower than 80% and higher or equal than 80%, respectively. ECOG-Index was 0 in 2%, 1 in 90%, and 2 in 8% of the patients. In total, 69% and 67% of the patients had a previous history of receiving enzalutamide and/or abiraterone and chemotherapy with docetaxel and/or cabazitaxel, respectively. Furthermore, distribution of the PSMA-avid metastases was as follows: bone only in 27.7% (*n* = 15) of the patients, local recurrence with lymph nodes in 14.8% (*n* = 8), bone and lymph nodes in 40.7% (*n* = 22), bone with hepatic and lymph node metastases in 9.5% (*n* = 5), and bone with pulmonary and lymph node metastases in 7.4% (*n* = 4) of the patients.

**Table 1:** Clinical characteristics of all studied mCRPC patients prior to PSMA-RLT therapy

Parameters	Values
Patients (*n*)	54
Age (mean ± SD)	72 ± 7 years
Weight (mean ± SD)	82 ± 13 kg
[^177^Lu]Lu-PSMA-617 (mean ± SD)	7315 ± 573 MBq
Karnofsky score (*n*)	
< 80% ≥ 80%	(18) 33 % (36) 67 %
ECOG-Index (*n*)	
0 1 2	(1) 2 % (49) 90 % (4) 8 %
Patients with previous enzalutamid/abirateron	69 %
Patients with previous docetaxel/cabazitaxel	67 %
Metastatic lesions (*n*)	
Bone Local recurrence + lymph node Bone + lymph node Bone + lymph node + liver Bone + lymph node + lung	(15) 27.7% (8) 14.8% (22) 40.7% (5) 9.2% (4) 7.4%

### Response and effects of PSMA RLT 1 month after the 3rd cycle

Among all treated patients, 79% (43 out of 54) of them showed PSA-response in terms of any PSA decline, whereas 58% and 35% presented with a PSA-decline of ≥ 50% and ≥ 80%, respectively. The median serum levels of PSA 1 month after receiving the last (3rd) cycle of Lu-PSMA RLT were significantly lower than before start of treatment (19.8 μg/L [range 0.7–4563] vs. 66 μg/L [range 1.0–4890], *P* < 0.001).

Generally, all patients tolerated the therapy very well and no acute adverse effects were reported during the hospital stay. Moreover, although serum levels of hemoglobin did not differ significantly before and after receiving 3 cycles of PSMA-RLT (11.7 ± 1.9 g/dL vs. 11.4 ± 1.9 g/dL, *P* = 0.11), levels of thrombocytes and leucocytes were significantly lower after the 3rd therapy than before therapy (236 g/L ± 71 vs. 193 g/L ± 67 and 6.5 g/L [range 2.9–13.7] vs. 4.8 g/L [range 1.5–12.3], *P* < 0.001), respectively. Nevertheless, no thrombocyte levels lower than 75 g/L and no leucocyte levels lower than 1.5 g/L were observed among the treated patients. In general, no grade 3 thrombocytopenia was observed, but 2 patients developed grade 3 leukocytopenia (leucocyte levels were between 1.9 and 1.5 g/L) and only one patient revealed grade 3 anemia (hemoglobin 6.9gm/dL) after the 3rd PSMA RLT. No grade 4 hematological toxicity occurred. No statistically significant differences were observed in levels of creatinine among the patients before and 1 month after receiving 3 cycles of PSMA-RLT. Laboratory parameters of all studied patients before and after PSMA-RLT are presented in Table 2.

**Table 2:** Laboratory parameters of mCRPC studied patients before and after 3 cycles of PSMA-RLT at 4 weekly intervals

Parameters	Before therapy	After therapy	*P* value
*PSA μg/L	66 (1.0–4890)	19.8 (0.7–4563)	< 0.001
Hemoglobin g/dL (mean ± SD)	11.7 ± 1.9	11.4 ± 1.9	NS
Thrombocytes g/L (mean ± SD)	236 ± 71	193 ± 67	< 0.001
*Leucocytes g/L	6.5 (2.9–13.7)	4.8 (1.5–12.3)	< 0.001
*Creatinine mg/dL	0.9 (0.6–1.6)	0.86 (0.6–1.8)	NS
*Alkaline phosphatase U/L (mean ± SD)	66 (27–469)	68 (27–580)	NS
*LDH (mean ± SD) U/L	189 (136–620)	197 (125–323)	NS

^*^Data with no Gaussian distribution, presented in median (range) and log_10_-transformd for analysis. *NS* not significant. *P* < 0.05 = statically significant

### Survival rate estimation

**Fig. 1:**
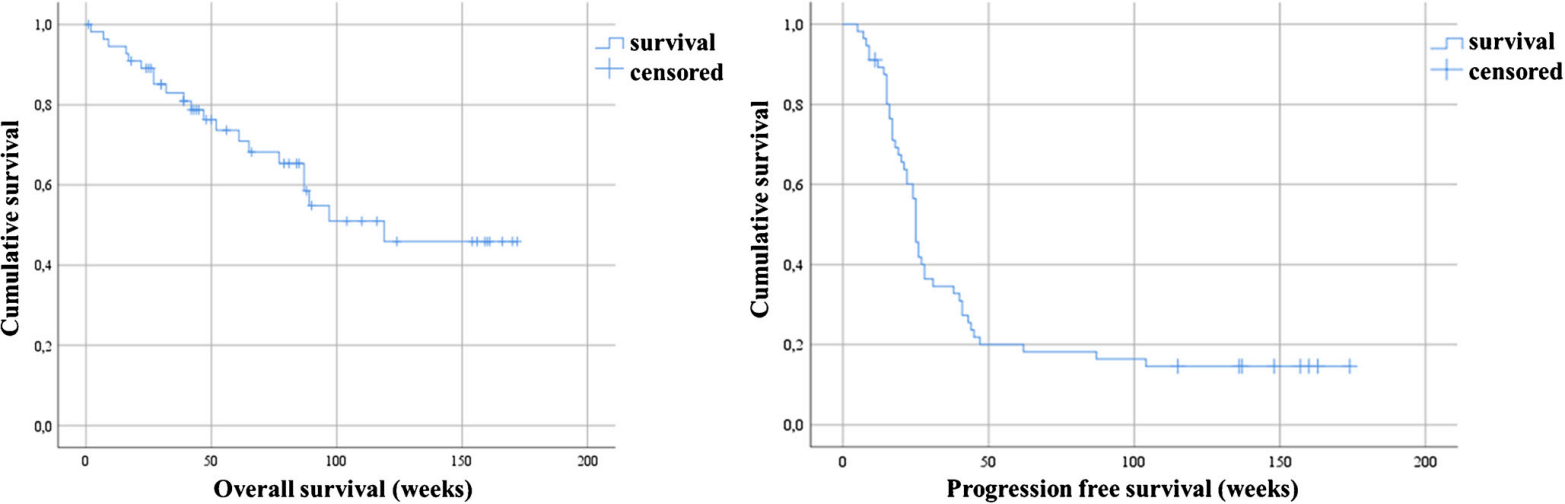
Kaplan-Meier plot shows median overall survival and median progression-free survival among the entire studied cohort. Patients received 3 cycles of highly standardized PSMA-RLT at 4 weekly intervals: The median overall survival was 119 weeks and the median progression-free survival was 25 weeks

**Fig. 2:**
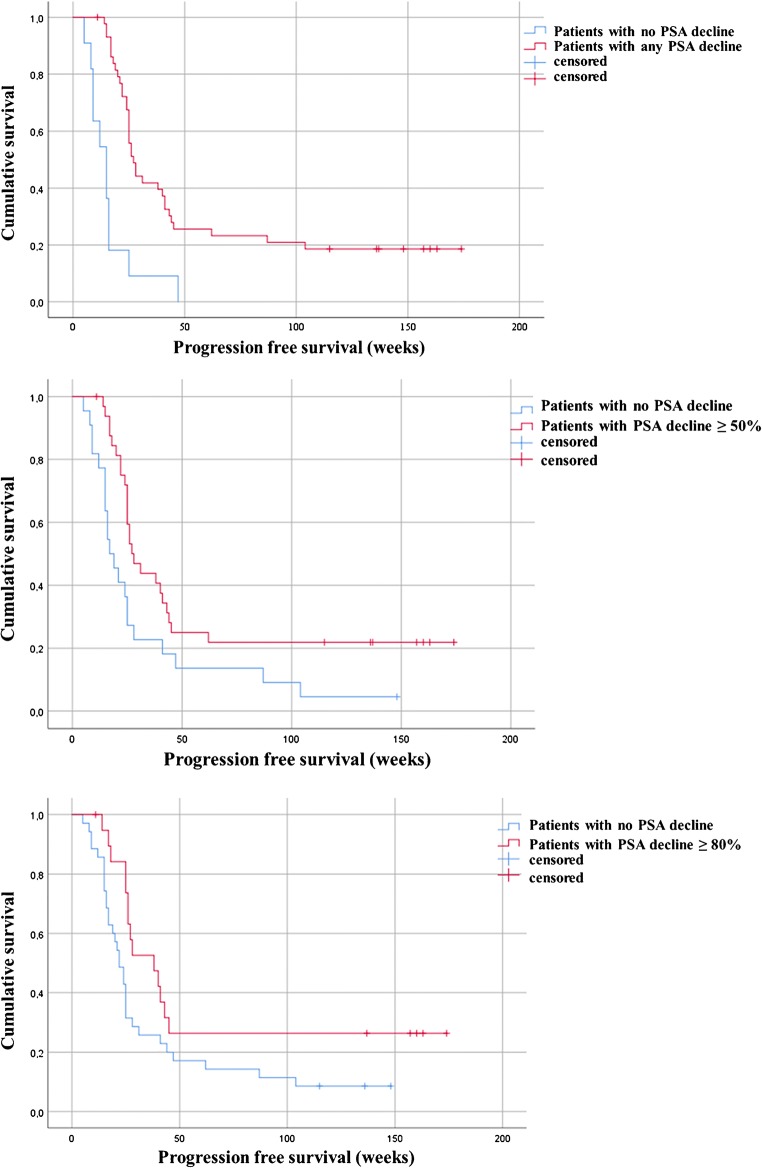
Kaplan-Meier plot shows progression free survival of the patients stratified by PSA response. Patients with any PSA reduction revealed significantly longer PFS than patients with no PSA decline (27 vs. 15 weeks, *P* < 0.0001). Patients with a PSA reduction of ≥ 80% had significantly longer PFS than patients with no PSA reduction (38 vs. 22 weeks, *P* = 0.03)

**Fig. 3:**
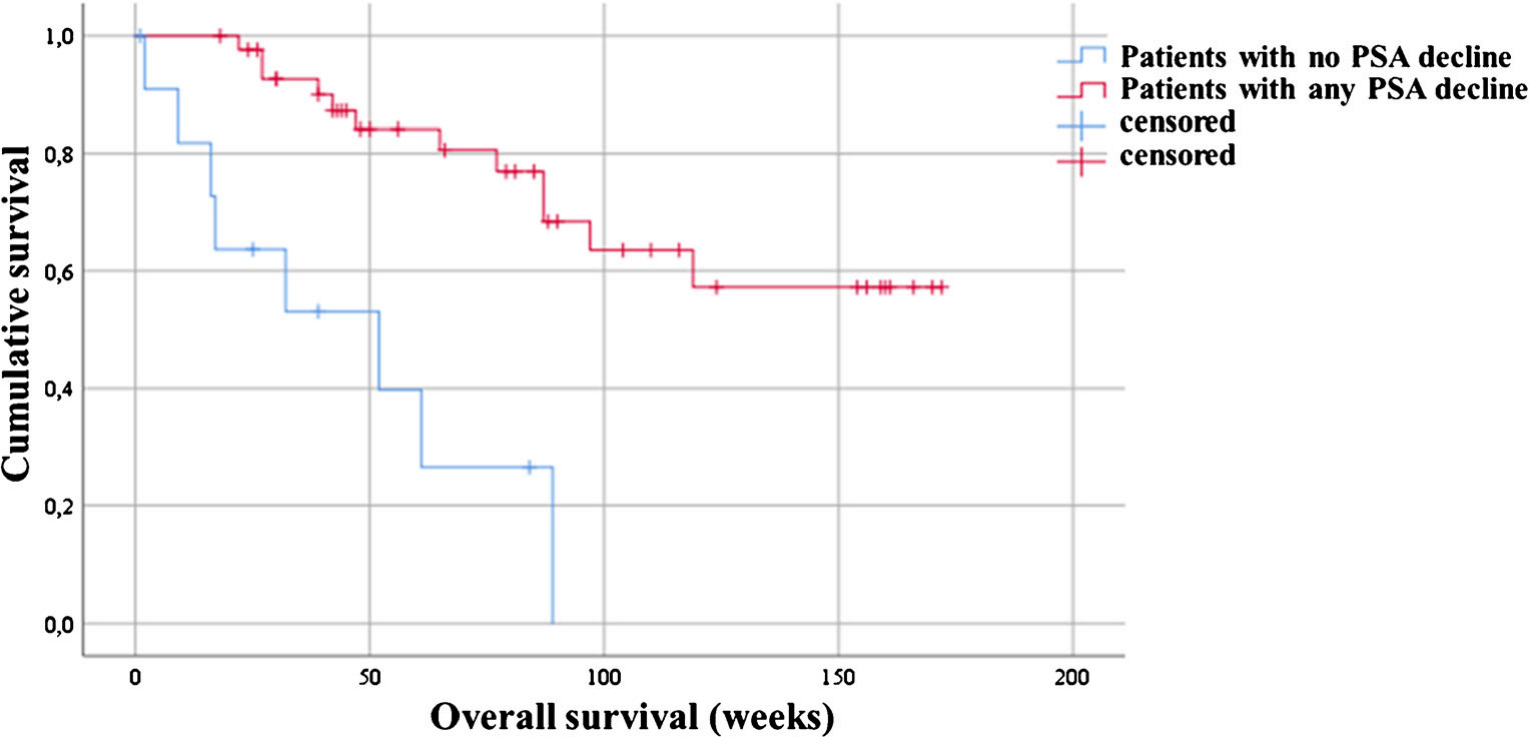
Kaplan-Meier survival plot of the patients stratified by PSA response. In comparison to patients with no PSA decline, patients with any PSA reduction up to 50% and more lived significantly longer (median survival not reached vs. 52 weeks, *P* < 0.0001)

**Fig. 4:**
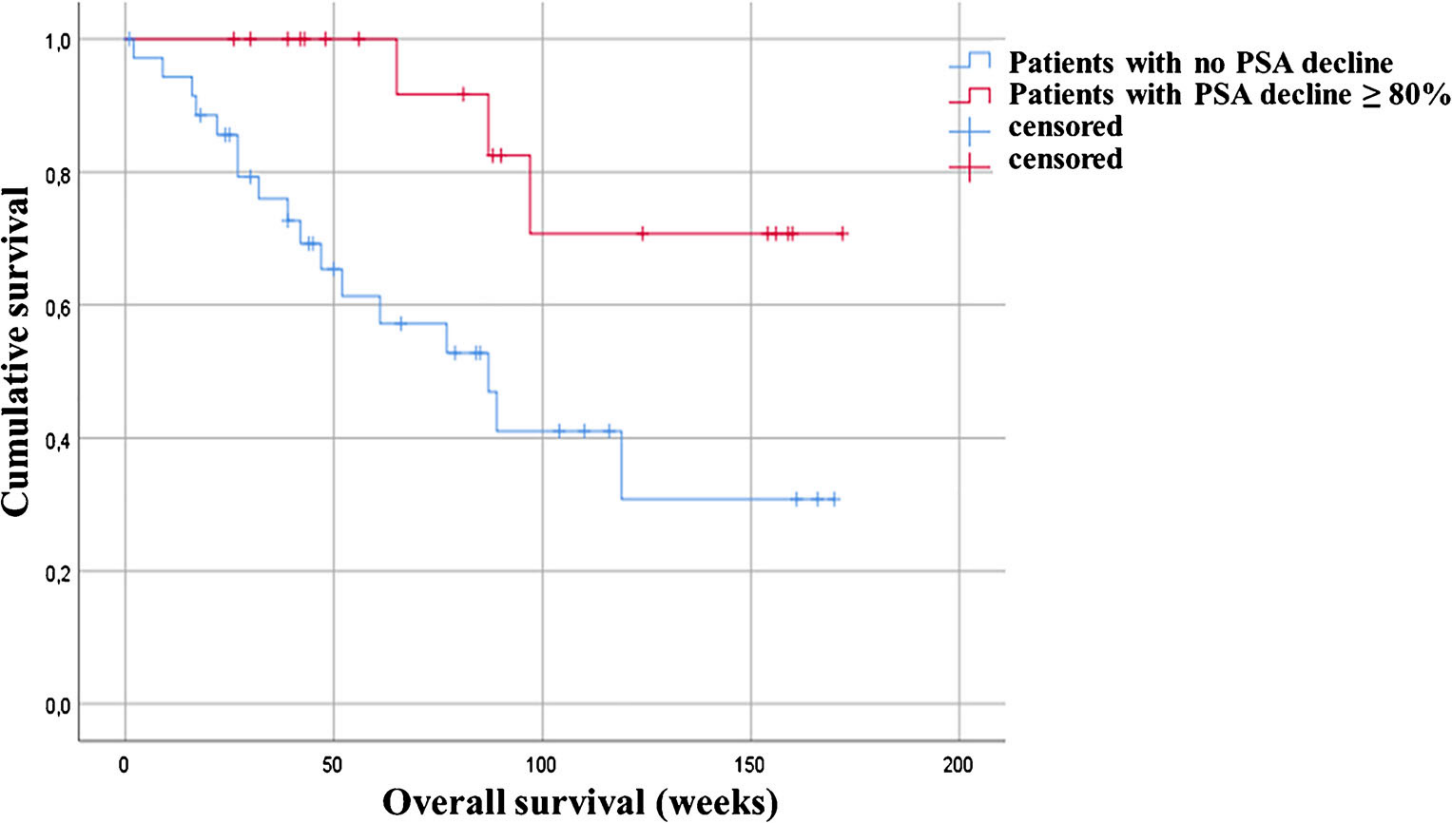
Kaplan-Meier survival plot of patients with a PSA drop ≥ 80%. Patients with a PSA reduction of ≥ 80% after therapy have significantly longer survival than patients with no PSA decrease (median survival not reached vs. 87 weeks, *P* = 0.008)

**Fig. 5:**
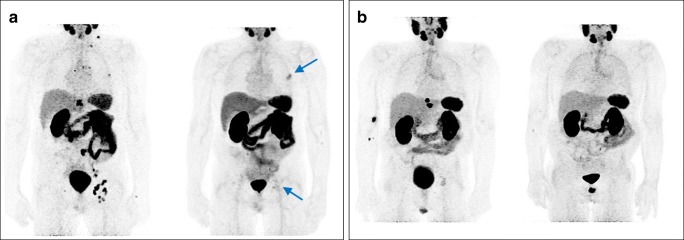
[68Ga]Ga-PSMA PET images of mCRPC patients before and after 3 cycles of PSMA-RLT: **a** patient with therapy response and PSA drop of about 70% (initial PSA 80.95 μg/L, after therapy PSA 22.95 μg/L); the arrows indicate residual metastatic areas. **b** Patient with therapy response and PSA decline of ≥80% (initial PSA 70.68 μg/L, after therapy PSA 6.4 μg/L)

The median OS among the entire treated cohort was 119 weeks (95%CI, 91.7–130.0) and the median PFS was 25 weeks (95%CI, 23.1–26.8) (Fig. 1). Moreover, results of Kaplan-Meier analysis indicated that patients with any PSA decline after 3 cycles of PSMA-RLT had a significantly longer PFS than patients with no PSA reduction (27 vs. 15 weeks, *P* < 0.0001). A decline of PSA of ≥ 50% and ≥ 80% was associated with increased PFS (≥ 50%, 27 weeks vs. 17 weeks, *P* = 0.01; ≥ 80%, 38 weeks vs. 22 weeks, *P* = 0.034) (all Fig. 2). Furthermore, patients with any PSA decrease showed a significantly longer OS than patients with increasing PSA (52 weeks vs. median survival not reached, *P* < 0.0001) (Fig. 3). Likewise, patients with reduction in PSA levels ≥ 50% (median survival not reached vs. 52 weeks (95%CI, 25.0–66.8), *P* < 0.0001) and a PSA reduction of ≥ 80% (median survival not reached vs. 87 weeks (95%CI, 68.4–115.8), *P* = 0.008) after the last cycle of the therapy lived significantly longer than patients with no PSA decrease (Figs. 3 and 4). Representative examples of therapy response with [^68^Ga]Ga-PSMA PET images are illustrated in Fig. 5.

## Discussion

The introduction of PSMA-RLT has further expanded the treatment options for patients with mCRPC [7]. In this study, we analyzed for the first-time data of mCRPC patients, the majority pretreated with chemo- and/or antihormonal therapies, and received 3 cycles of highly standardized PSMA-RLT at 4 weekly intervals. We mainly concentrated on the safety as well as efficacy, rate of response, and the clinical outcome of this therapy protocol among the treated patients. Although, there is an ongoing approach to standardize PSMA-RLT protocols [12], the recent meta-analysis of Yadav et al. [9] has briefly summarized data of several PSMA-RTL studies and showed inhomogeneity of in these studies concerning doses and intervals of the provided therapies. Up to the present, there is no information about safety and effectivity of the PSMA-RLT in mCRPC patients received equally high-dose of the therapy with merely 4 weeks interval between the cycles and, to the best of our knowledge, we are, at the moment, the only clinical center applying this therapeutic protocol. The rationales for developing such a protocol were, on the one hand, the promising results of safety and toxicity with an approximately 6.0 GBq every 8 weeks protocol from another working group [13] and on the other hand, the hypothesis that a follow-up therapy after circa 4 half-lives of ^177^Lutetium will keep up the effective dose in the tumor more efficiently than an 8-week gap, following a concept similar to a fractionated radiation therapy. The results of our study revealed response rates to the PSMA-RLT in terms of any PSA decline in 79% of the treated cases and 58% and 35% of them presented with a PSA-decline of ≥ 50% and ≥ 80%, respectively. Probably because of differences in degrees of tumor stage among the treated patients and differences in applied therapies prior the PSMA-RLT, this considerable finding is slightly superior to results of previous studies that have demonstrated response rates to PSMA-RLT of about 60–70% in the treated mCRPC patients [4, 6, 14–16]. Nevertheless, this result is in line with results of a previous phase II trial study that included 30 mCRPC patients with progressed disease treated with PSMA-RLT at 6 weekly interval after failure of the standard treatments and indicated a PSA reduction of ≥ 50% in 57% among the studied participants [10]. Furthermore, in the study of Emmett et al. that included 18 mCRPC patients receiving a mean dose of 7.0 GBq PSMA-RLT with 6 weeks interval between the cycles, a therapy response in terms of PSA reduction in 71% of the treated patients was reported [17]. In 36% of them, there was a reduction in PSA level of ≥ 50%. In Baum et al. [18], rate of response could be seen in 80% (45 out of 56) of the treated patients; here, the numbers of the applied cycles were very heterogeneous and varied from 1 to 5 cycles and the interval between the cycles was not defined.

Moreover, we observed no significant differences in serum hemoglobin levels before and after receiving 3 cycles of Lu-PSMA-RLT and although thrombocytes and leucocytes levels were significantly lower after the therapy, only two cases developed grade 3 leukocytopenia and no grade 3 thrombocytopenia was observed among the patients. Additionally, no relevant nephrological effects were noticed among the patients before and 1 month after receiving 3 cycles of PSMA-RLT. Similarly, various previously published studies indicated either low, minimal, or reversible hemato- and nephrotoxicity effects of PSMA-RLT in the treated patients [5, 18, 19]. Ahmadzadehfar et al., in their first experience with PSMA-RLT, reported early side-effects in terms of some degrees of hematotoxicity in only few treated cases without evidence of relevant nephro- or hepatotoxicity 8 weeks after receiving a single dose of the therapy [4]. In another study, they could confirm safety of PSMA-RLT in 24 mCRPC patients obtaining repeated cycles of the therapy [14].

In addition, we found that the median OS for the entire treated patients was 119 weeks and the median PFS was 25 weeks. Rahbar et al. showed recently a median OS of 56 weeks in the entire 104 mCRPC patients who received an inhomogeneous number of PSMA-RLT ranged between 1 and 8 cycles at 8 weeks interval [16]. This notable difference in patient’s median OS between these 2 studies might either reflect the disparity of the patient’s population and their tumor burden as our study included almost 30% chemotherapy naïve patients, while in Rahbar et al., the entire studied cohort was previously treated with chemotherapy or points towards a superior efficacy of high doses of 3 cycles PSMA-RTL in short 4-week interval. Furthermore, it has previously been demonstrated that administrations of repeated therapy cycles will increase the rate of response to the PSMA-RLT and this might consequently increase the OS, as about one third of treated mCRPC patients reveal late therapy response [20]. However, we (Fig. 3) and Rahbar et al. could observe a nearly identical longer survival among patients exhibiting any PSA decline than patients with PSA progression [16]. Similarly, depending on presence and the degree of PSA decline after the therapy, our and other studies could show that PFS and OS are significantly longer in responding patients [5, 7, 10, 21, 22]. The observations of very low toxicity and comparatively high response rates among treated patients with this presented therapeutic scheme might enable treatments with even higher PSMA-RLT doses, as this has also been shown by Rathke et al. [23] and could encourage systematic dose-finding studies for this treatment.

Although this study included a homogeneous group of mCRPC patients treated with a homogeneous scheme of PSMA-RLT, the retrospective design is the main limitation of this study. Moreover, degrees of therapy toxicity observed among patients included in this study might have been affected by the differences in patient’s population in terms of pre-treatment and disparities in tumor stage. Therefore, further larger prospective studies with a more homogeneous patient population using this promising therapy regime are essential to confirm our results and to move forward towards global standardization of this therapy in patients with mCRPC.

## Conclusion

Results of this study showed that mCRPC patients treated with a highly standardized PSMA-RLT regime at four weekly intervals routinely applied in our clinic have a considerable response rate and a favorable PFS and survival. In addition, results demonstrated good tolerability of PSMA-RLT.
